# Transmission Channels and Impacts of Energy Use on Health Outcomes in Asia

**DOI:** 10.3389/fpubh.2021.811872

**Published:** 2022-01-12

**Authors:** Xiaoyan Zhang, Minjuan Chen, Jinbao Li

**Affiliations:** ^1^Business School, Shandong Normal University, Jinan, China; ^2^School of Economics, Guangxi University for Nationalities, Nanning, China

**Keywords:** energy use, health, Asia, human, life expectancy

## Abstract

Today, the developing economies continue to tackle the penalties of the energy use and its influence on their environmental and socio-economic prosperity, and the developed economies are concentrating on promoting programs and policies to improve and sustain the endowment of adequate energy consumption that pledges less carbon emissions and threats to human health. Currently, millions of people face a dearth of access to reliable, affordable, and clean energy to fulfill their daily requirements. Thus, the mounting need for energy use portends hazardous consequences on human health. This paper investigates the transmission channels and impact of energy consumption on health outcomes in Asia by adopting a panel of selected Asian economies for the period from 1991 and 2019. The findings of the study show that energy causes a rise in infant mortality rate and a reduction in life expectancy. Furthermore, the study found that a high degree of pollution emissions causes a rise in infant mortality and a decline in life expectancy.

## Introduction

The developing economies, in recent times, have dual challenges in front of them; the first one is to speed up their process of economic development; secondly, they rely heavily on non-renewable energy sources such as coal, oil, and gas to lift the living standards of the people (1). The consumption of renewable energy sources is proved to be the engine of economic growth and helps to grease the wheel of the economy (2). On the other side, non-renewable energy sources are the major reason behind greenhouse gas (GHG) emissions and climate change. Consequently, GHG emissions are the biggest threat to ecological and human health (3). Developing and emerging economies follow one common economic goal, i.e., development. They cannot afford to compromise on this goal because to raise the living standards of their people, rapid economic growth is essential (4). Individuals and firms in these developing economies rely heavily on non-renewable energy sources to fulfill their energy demand by ignoring the harmful effects of non-renewable energy consumption on the environment and human health (5).

The role of energy is very significant in achieving economic growth and development because economic development and energy consumption go up side by side and economic growth is highly dependent on energy consumption (6). In recent times, sustainable and clean energy production has become one of the most challenging for developing and emerging world (7). Several pieces of evidence are available, which confirm that inhalable particulate matter (PM), methane, micro metal elements, and SO_2_ are among the topmost pollutants that negatively affect environmental and human health. Incomplete energy combustion is one of the biggest reasons for increasing air pollution, a major source of respiratory diseases in children and elders in developing economies. Consequently, each year, 43 million people around the globe die because of respiratory diseases. Respiratory infections have become a serious threat to the lives of all age groups of the society; therefore, health policies in many developing and emerging economies are particularly focusing on and targeting the diseases related to the respiratory system (8).

Since the 1980s, the relationship between energy consumption and health outcomes has gathered many environmentalists' and policy makers' attention. Most past studies have tried to analyze how technological innovations and energy-related policies could help mitigate the degradation in the environment and public health. The nexus between energy consumption, environment, and public health has got popularity in recent times, particularly in the economies like China, India, and Thailand. As a result, the Triple Carbon-Balance analysis method of Smith has been extensively used in these types of researches (9). Many studies have not directly analyzed the relationship between energy consumption and human health. However, most studies have examined the said relationship by including the environment variable and focused on the energy, environment, and human health nexus (10). By using the theory of “Total exposure assessment,” Smith tried to examine the position of pollution in China (11). Smith introduced the exposure assessment method and analyzed the impact of energy consumption on air quality and air quality on human health (9).

Künzli et al. (12) gathered the data on three European countries (Austria, France, and Switzer) and tried to examine the impact of traffic-driven air pollution on the mortality and morbidity rate by using “Epidemiology-based exposure-response functions.” Clancy et al. (13) analyzed the role of energy substitution in lowering the mortality rate in light of the policy of banning coal sales in Dublin. They gathered the data before and after the imposition of a ban and found that deaths caused by pollution significantly reduced after the imposition of the ban. Ezzati et al. (14) analyzed the role of introducing sophisticated and modern energy technologies in society and their impact on human health. The result showed that during 2 years of introducing such technologies, the health of residents improved significantly. Particularly, improving stove efficiency has significantly reduced the mortality rate and cut the rate of breathing infections in children. One of the widely used methods in the determination of energy-environment-health nexus in economics is cost-effective analysis (15). On the other side, the metrology method is a way for measuring the cost-effective analysis, which is widely used in the “Energy–Environment–Health” in the field of economics to calculate the preventable health damages instigated by a decrease in carbon discharges and the connected cost in the coming years (16). To measure the difference between cost and benefit, a method of calculating the net economic cost of diverse energy policies can be used (17). Tsikalakis and Hatziargyriou (18), by using the emission trading markets, applied the cost and benefit principles to measure the cost of decreasing pollutant emissions (19). The findings of this study confirmed that the cost of controlling emission varies across different regions and countries. Normally, the cost of controlling pollution emission is higher in developing economies.

In light of the above arguments, we can now confirm that very few studies have directly captured the impact of energy consumption on health outcomes in Asian economies. Therefore, to the best of our knowledge, we can say that this is the first-ever study that has tried to analyze the energy consumption and health outcomes nexus, particularly in the context of Asian economies. Empirical outcomes should enable policymakers to produce more useful energy and health policies. This study provides some new insights into environmental pollution to strengthen health systems and energy policy. The current study is highly important in the energy and health sectors for policymakers.

The organization of the study is as follows. The next section covers the model and methods, followed by the material in section three. In section four, results are provided, and section five illustrates the conclusion.

## Model and Methods

A pioneer work of Grossman (20) that recognized the health outcomes function model, Shobande (21) improved the theory. The health outcomes function describes the relationship between environment-related factors that are affecting health status. Previous empirical studies Hanif, Youssef et al., Pesaran et al. (5, 22, 23) intended the work and noted that energy consumption significantly impacts mortality rates and life expectancy. The theoretical and empirical literature has confirmed that energy consumption greatly impacted the quality of life and life expectancy. This study has examined the effect of energy use on health outcomes in Asia. To achieve the objective, we have followed an empirical model by (22). The basic form is:


$$\begin{array}{l} Health_{\;it}=\;\varphi_{1}+\;\varphi_{1}EC_{it}+\varphi_{2}CO_{2,it}+\varphi_{3}GDP_{it}+\varphi_{4}HE_{it}\\ +\varphi_{5}Trade_{it}+\varphi_{6}UP_{it}+\varepsilon_{it} \end{array}$$

Health, EC, CO_2_, GDP, HE, Trade, and UP represent health outcomes that are infant mortality and life expectancy, energy consumption, CO_2_ emissions, GDP per capita, health expenditure, trade openness, and urban population, respectively. In the panel model, i stated country, and *t* signifies the data period. In model, φ_1_, φ_2_, φ_3_, φ_4_, φ_5_, φ_6_ are the elasticities of healths outcomes with respect to energy consumption, CO_2_ emissions, GDP per capita, health expenditure, trade openness, and urban population. Empirical evidence noted that energy consumption has a negative effect on health outcomes and an estimate of φ_1_ is expected to be negative. While α_*i*_ is unobservable individual effects, and εit is the error terms in the model. The extended form is:


$$\begin{array}{l} Health_{\;it}=\;\varphi_{1}+\;\varphi_{1}EC_{it}+\varphi_{2}CO_{2,it}+\varphi_{3}GDP_{it}+\varphi_{4}HE_{it}\\ +\varphi_{5}Trade_{it}+\varphi_{6}UP_{it}+\alpha_{i}+\varepsilon_{it} \end{array}$$

In the panel model, firstly, we have confirmed cross-sectional dependence in data. We also used Im et al. (24) test for the cross-sectional dependence (CD) test in panel data. This test uses the cross-correlation between the time-series for each economy in panel data. The second step is to confirm whether concern variables are integrated of the same order. There are several unit roots tests in panel data, but this study has also employed Im, Pesaran and Shin (IPS) unit root tests (25). These unit root tests are adjusted cross-sectional dependence in analysis. The next step is to test for panel cointegration if all concern variables are integrated in the same order that assesses the long-run relationship among the variables. In the next step, we examine whether a long-run relationship exists among concern variables.

This study has employed FMOLS proposed by Pedroni (26) and Kao and Chiang (27) and the DOLS methods proposed by Zafar et al. (28) that were used to strengthen the long-term estimates. These estimation methods are more powerful than the OLS estimator. Although FMOLS is one of the parametric methods that results by correcting serial correlation errors and endogenous, DOLS is a non-parametric method (29). However, one of the common features of FMOLS and DOLS methods is that they can overcome the problems of small sample bias and endogenous (30). Both methods are the workhorse of panel data in the modern era. The two approaches yield similar outcomes for each variable in terms of sign and significance but slightly vary in terms of magnitude.

## Data

The study aims to explore the transmission channels and influence of energy consumption on health outcomes in the case of selected Asian economies. These economies include Bangladesh, China, India, Indonesia, Malaysia, Philippines, Sri Lanka, and Thailand. These economies are selected based on the availability of data. Health outcome is measured by infant mortality (i.e., measured as mortality rate per 1,000 live births) and life expectancy (i.e., measured as life expectancy at birth in total years). Energy use is measured as energy consumption (kg of oil equivalent per capita) and fossil fuel energy consumption (percentage of total energy consumption). The study incorporated the role of CO_2_ emissions (kilotons), GDP per capita (constant 2015 US$), current health expenditure (percent of GDP), trade openness (percent of GDP), and urban population (percentage of the total population) as control variables. For empirical investigation, data on all variables are extracted from the World Bank, and details are given in Table 1.

**Table 1:** Data and definitions.

**Variables**	**Symbol**	**Definitions**	**Sources**
Infant mortality	Minf	Mortality rate, infant (per 1,000 live births)	World bank
Life expectancy	LE	Life expectancy at birth, total (years)	World bank
Energy consumption	EC	Energy use (kg of oil equivalent per capita)	World bank
Fossil fuel energy consumption	FEC	Fossil fuel energy consumption (% of total)	World bank
CO_2_ emissions	CO_2_	CO_2_ emissions (kt)	World bank
GDP per capita	GDP	GDP per capita (constant 2015 US$)	World bank
Health expenditure	HE	Current health expenditure (% of GDP)	World bank
Trade openness	Trade	Trade (% of GDP)	World bank
Urban population	UP	Urban population (% of total population)	World bank

## Results and Discussion

As a first step, the study investigates the cross-sectional dependence of the panel data. In Table 2, the finding implies that cross-sectional dependence exists among Asian regions. Before performing regression analysis, the study performed unit root tests to confirm the stationarity properties of data. For that purpose, the study employed cross-sectional dependence IPS unit root tests. Table 3 displays the empirical outcomes of these three tests. Findings demonstrate that all variables possess first difference stationarity properties, i.e., hold I(1) order of integration. However, none of the variables is stationary at the second difference. At the same time, the validation of cointegration indicates the existence of a long-run association among all study variables in Table 4.

**Table 2:** Cross-sectional dependence tests.

**Model-Mnf**	**Minf**	**EC**	**CO_**2**_**	**GDP**	**HE**	**Trade**	**UP**
Pesaran CD test	−1.673^*^	−5.710^***^	0.213	−0.075	−2.090^**^	4.374^***^	−1.466
*P*-value	0.094	0.000	0.831	0.940	0.036	0.000	0.142
**Model-LE**	**LE**	**EC**	**CO**_**2**_	**GDP**	**HE**	**Trade**	**UP**
Pesaran CD test	−5.928^***^	−2.235^**^	−1.795^*^	−0.081	−2.001^**^	4.469^***^	−2.018^**^
*P*-value	0.000	0.025	0.072	0.985	0.045	0.000	0.043

*** *p < 0.01*;

** *p < 0.05; and*

* *p < 0.1*.

**Table 3:** CIPS unit root test.

	**Minf**	**LE**	**EC**	**CO_**2**_**	**GDP**	**HE**	**Trade**	**UP**
I(0)	−0.035	−0.307	−0.847	−0.582	0.568	−0.830	0.051	−0.753
I(1)	−4.855^***^	−3.526^***^	−7.695^***^	−7.043^***^	−4.405^***^	−8.746^***^	−7.200^***^	−2.191^**^
Decision	I(1)	I(1)	I(1)	I(1)	I(1)	I(1)	I(1)	I(1)

*** *p < 0.01 and*

** *p < 0.05*.

**Table 4:** Pedroni panel cointegration test results.

	**Minf**		**LE**	
	**Statistic**	***p*-value**	**Statistic**	***p*-value**
Common AR coefs. (within-dimension)
Panel v-statistic	−3.218^***^	0.000	−3.199^***^	0.000
Panel rho-statistic	1.937^**^	0.026	3.229^***^	0.000
Panel PP-statistic	−3.766^***^	0.000	1.706^**^	0.044
Panel ADF-statistic	−3.907^***^	0.000	1.383^*^	0.083

*** *p < 0.01*;

** *p < 0.05; and*

* *p < 0.1*.

For basic models regression analysis and variable-based robustness testing, the study adopted the FMOLS approach. However, for method-based robustness testing, the study employed the DOLS technique. Table 5 displays the empirical findings of group-wise models, and Table 6 displays the outcome of economy-wise models. In both tables, the study performed six independent regressions. In regression one, column 1, energy consumption impact on infant mortality rate is tested. In regression 2, column 2, energy use impact on life expectancy variable is determined. In regression 3, column 3, the association between energy use and infant mortality is measured by using the DOLS approach. In regression 4, column 4, the nexus between energy use and life expectancy is explored. In regression 5, column 5, fossil fuel energy consumption impact on infant mortality rate is examined. In the last regression, in column 6, the impact of fossil fuel consumption on life expectancy is examined.

**Table 5:** FMOLS estimates (group-wise).

	**Basic models (FMOLS)**		**Robustness with methods (DOLS)**		**Robustness with variables (FMOLS)**	
	**Minf**	**LE**	**Minf**	**LE**	**Minf**	**LE**
	**(1)**	**(2)**	**(3)**	**(4)**	**(5)**	**(6)**
EC	0.24^***^	−0.13^***^	0.16^***^	−0.11^***^		
	(9.04)	(10.6)	(3.55)	(6.18)		
FEC					0.33^***^	−0.16^***^
					(4.32)	(3.48)
CO_2_	0.03^***^	−0.04^***^	0.32^***^	−0.04^***^	0.23^***^	−0.02^***^
	(10.9)	(8.72)	(4.67)	(6.25)	(4.03)	(12.7)
GDP	−0.05^***^	0.01^***^	−0.85^**^	0.12^***^	−0.02^***^	0.07^***^
	(7.76)	(4.38)	(3.29)	(7.56)	(2.92)	(4.09)
HE	−0.06^***^	0.01^***^	−0.49^***^	0.04^***^	−0.04^**^	0.02^***^
	(3.39)	(8.16)	(8.05)	(7.75)	(2.24)	(8.99)
Trade	−0.01^***^	0.01^***^	−0.01^***^	0.01^***^	−0.05^***^	0.01^***^
	(8.57)	(7.47)	(3.36)	(6.59)	(6.28)	(7.12)
UP	−2.02^***^	0.03^***^	−2.06^***^	2.28^***^	−2.02^***^	0.01^***^
	(5.53)	(4.74)	(3.44)	(8.86)	(8.46)	(2.25)

*** *p < 0.01 and*

** *p < 0.05*.

**Table 6:** FMOLS estimates (Economy-wise).

		**EC**	**CO_**2**_**	**GDP**	**HE**	**Trade**	**UP**
Model-Minf	Bangladesh	0.44^***^	0.34^***^	0.05^*^	0.02	−0.02^***^	−1.57^***^
		(4.37)	(4.66)	(1.76)	(0.34)	(3.35)	(9.82)
	Malaysia	0.17	0.55^***^	0.91^***^	−0.02	−0.15^***^	−1.42^***^
		(1.51)	(5.67)	(11.7)	(0.54)	(4.05)	(3.93)
	Philippines	0.01	0.18^***^	−0.37^***^	0.04	−0.06^***^	4.27^***^
		(0.27)	(10.4)	(11.4)	(0.23)	(5.92)	(3.74)
	Thailand	1.37^***^	0.71^***^	−0.29	−0.33^***^	−0.15^*^	0.22
		(4.44)	(3.22)	(1.07)	(5.86)	(1.92)	(0.81)
	Sri Lanka	0.19^***^	0.17^***^	−0.76^***^	0.01	0.19^***^	−8.98^***^
		(5.39)	(12.7)	(6.76)	(1.27)	(14.2)	(8.31)
	China	1.39^***^	0.56^***^	0.59^***^	−0.07^***^	0.19^***^	−2.81^***^
		(3.23)	(11.9)	(8.16)	(8.82)	(11.5)	(15.6)
	India	0.12^***^	0.05^***^	−0.05^***^	−0.03^***^	0.06^***^	−3.88^***^
		(11.0)	(5.88)	(4.67)	(3.55)	(4.72)	(9.15)
	Indonesia	0.48^***^	0.16	−0.43^***^	0.01	0.06^*^	−2.03^***^
		(2.63)	(1.57)	(7.41)	(0.19)	(1.85)	(7.41)
Model-LE	Bangladesh	−0.21^***^	−0.17^***^	0.07^***^	0.01^***^	0.02^***^	0.07^**^
		(15.3)	(8.38)	(5.66)	(2.83)	(6.76)	(2.02)
	Malaysia	0.01	−0.01^**^	0.02^***^	0.01^*^	−0.02^***^	0.13^***^
		(1.08)	(2.24)	(4.73)	(1.92)	(11.9)	(8.23)
	Philippines	−0.01^***^	−0.03^***^	0.02^***^	0.01^***^	−0.01^***^	−0.16^***^
		(3.85)	(8.93)	(9.74)	(5.93)	(10.2)	(8.79)
	Thailand	−0.06^***^	−0.06^***^	0.02	0.01^***^	−0.02^***^	0.12^***^
		(2.74)	(3.52)	(1.12)	(2.49)	(3.25)	(5.82)
	Sri Lanka	−0.15^***^	0.01	0.03^*^	0.01^*^	−0.02^*^	−0.92^***^
		(4.65)	(0.41)	(1.71)	(1.71)	(1.84)	(5.42)
	China	−0.03^***^	−0.02^***^	0.03^***^	0.01^***^	0.01^***^	0.22^***^
		(8.85)	(10.3)	(4.53)	(6.69)	(8.48)	(6.16)
	India	−0.24^***^	−0.22^***^	0.01	0.01	0.01^***^	0.04
		(9.63)	(4.79)	(0.91)	(0.01)	(9.39)	(1.64)
	Indonesia	−0.06^***^	−0.02^**^	0.06^***^	0.01	0.01^**^	0.23^***^
		(4.63)	(2.39)	(14.3)	(1.54)	(2.01)	(11.9)

*** *p < 0.01*;

** *p < 0.05; and*

* *p < 0.1*.

The empirical findings of the basic model (group-wise) reveal that energy consumption produces a significant and positive impact on infant mortality rate, revealing that due to an increase in energy use, the infant mortality rate rises. Coefficient estimates infer that in response to a 1 percent upsurge in energy use, the infant mortality rate rises by 0.24 percent. In terms of control variables, findings display that CO_2_ emissions result in an increasing infant mortality rate. However, GDP per capita, trade openness, health expenditures, and urban population result in reducing the infant mortality rate. In the case of the life expectancy model (column 2), findings show that energy use results in producing a significant and negative impact on life expectancy, confirming that due to an upsurge in energy consumption, the life expectancy of people tends to decline. The coefficient estimate shows that due to a 1 percent rise in energy consumption, life expectancy declines by 0.13 percent. CO_2_ emissions produce significant and negative impacts, revealing that life expectancy tends to decline due to the increase in pollution emissions. In contrast, increased other control variables, such as GDP per capita, health expenditures, trade openness, and urbanization, increased life expectancy. The study confirms the robustness of findings by technique exchange method (columns 3 and 4) and variable exchange method (columns 5 and 6). The study found similar kinds of findings from both methods of robustness.

In the existing stock of literature, the Gary production theoretical framework explains the linkage between consumption and production of energy sources and its influence on infant health risks. This study is also reliable with Zelenáková et al. (31), who explored the spatial association between different sources of energy utilization and life expectancy in China by adopting a spatial correlation method. It is concluded that the use of biomass adversely influences life expectancy in China. Likewise, Lobel et al. (32) evaluated and examined small-scale hydropower plants' influence on the environment by adopting a quantitative evaluation method and investigated the impact on the social life and health of the population. People of Aian economies used to consume readily accessible sources of energy in the form of gas, coal, and oil (i.e., fossil fuels) and crop residues, wood pellets, wood, peat, and charcoal (i.e., solid fuels) to fulfill their indoor and outdoor energy needs, overseeing the aggressive influence of poisonous gases on the health of people (33).

The massive consumption of fossil and solid fuels required to fulfill energy needs is increasing, and this is establishing both economic and health-related issues. To fulfill intensifying energy demand, these economies are consuming fossil fuel in larger quantities, and product wastage in the form of harmful gases, which in turn adversely affect the health condition of people and especially contribute to intensifying pulmonary diseases such as asthma and tuberculosis, and above all these practices intensifies environmental issues. The findings of our study are in line with the empirical outcomes of Sapkota (34) and Machol and Rizk (35), endorsing that consumption of energy and fossil fuel consumption adversely affects health conditions in the sample of selected Asian economies. The findings also reveal that the use of solid for cooking purposes directly injects toxic gases into the environment, such as nitrogen oxides (NOx) and carbon monoxide (CO), which ultimately generate serious health issues. These findings validate the empirical outcomes of (36) and (37). The combustion of coal and biomass in urban regions tends to a high degree of outdoor and indoor pollution that harms health conditions. Our study findings discourage the consumption of solid and fossil fuels to fulfill the indoor and outdoor energy needs, highlighting the significance of economic development and renewable energy to enhance life expectancy and reduce infant mortality by controlling the health issues in selected Asian economies.

Table 6 displays the empirical outcomes of economy-wise estimates. The findings show that energy use has a significant and positive impact on the infant mortality rate in all selected Asian economies except Malaysia and the Philippines, confirming that an increase in energy consumption increases the infant mortality rate. Coefficient estimates show that due to a 1 percent rise in energy use, the infant mortality rate increases by 0.44 percent in Bangladesh, 1.37 percent in Thailand, 0.19 percent in Sri Lanka, 1.39 percent in China, 0.12 percent in India, and 0.48 percent in Indonesia. An increase in CO_2_ emissions tends to increase the infant mortality rate in all Asian economies except Indonesia. GDP per capita results in an increasing infant mortality rate in Bangladesh, Malaysia, and China and significantly decreases infant mortality rate in the Philippines, Sri Lanka, India, and Indonesia. An increase in health expenditure results in a declining infant mortality rate in Thailand, China, and India. Trade openness decreases the infant mortality rate in Bangladesh, Malaysia, the Philippines, and Thailand, increasing infant mortality rates in China, Sri Lanka, India, and Indonesia. Urbanization significantly declines the infant mortality rate in Bangladesh, Malaysia, China, Sri Lanka, India, Indonesia and significantly increases the infant mortality rate in the Philippines.

In the life expectancy model, findings disclose that increase in energy use produces a significant and negative impact on life expectancy in all selected Asian economies except Malaysia. In response to a 1 percent upsurge in energy use, it shows that life expectancy declines by 0.21 percent in Bangladesh, 0.01 percent in the Philippines, 0.06 percent in Thailand, 0.15 percent in Sri Lanka, 0.03 percent in China, 0.24 percent in India, and 0.06 percent in Indonesia. An increase in CO_2_ emissions tends to decline life expectancy in all selected economies except Sri Lanka. An increase in GDP per capita results in increasing life expectancy in all selected economies except Thailand and India. Findings show that life expectancy increases in all selected economies except India and Indonesia due to an increase in health expenditures. Trade openness results in increasing life expectancy in Bangladesh, China, India, and Indonesia and tends to decline life expectancy in the rest of the economies. Urbanization leads to a significant increase in life expectancy in Bangladesh, Malaysia, Thailand, China, and Indonesia and declines in life expectancy in the Philippines and Sri Lanka.

## Conclusion and Implications

The study investigated the transmission channels and energy consumption influence on the health condition of selected Asian economies such as Bangladesh, China, India, Indonesia, Malaysia, Philippines, Sri Lanka, and Thailand. The study adopted a variable-based approach and technique-based approach to check the robustness of findings. In this regard, the study used two proxies to measure health outcomes these are life expectancy and infant mortality rate. Energy use is also measured by two proxies such as energy consumption and fossil fuel energy consumption. The study employed the FMOLS technique for basic regression analysis and employed the DOLS approach for robustness testing. Moreover, the study incorporated some important control variables for analysis such as CO_2_ emissions, GDP per capita, trade openness, health expenditures, and urban population. The group-wise findings suggest that energy consumption tends to increase infant mortality rate and reduce life expectancy. In terms of CO_2_ emissions, findings demonstrate that an increase in CO_2_ emissions results in an increasing infant mortality rate and reduced life expectancy. In contrast, an increase in GDP per capita, trade openness, health expenditures, and urban population increases infant mortality rate and declines life expectancy. Findings of robust models are also in line with the findings of basic models. The economy-wise findings reveal that increase in energy consumption results in an increasing infant mortality rate in Bangladesh, China, India, Indonesia, Sri Lanka, and Thailand and reduce life expectancy in Bangladesh, China, India, Indonesia, Philippines, Sri Lanka, and Thailand. Findings show that an increase in CO_2_ emissions contributes adversely to increasing mortality rate and declining life expectancy in mostly Asian economies. In the case of other control variables, findings suggest a favorable impact on infant mortality rate and life expectancy in mostly selected Asian economies.

On the policy side, governments, energy planners, and health economists' associated bodies must act together in implementing strategies for energy and health sectors deployment across countries. To reduce the negative impact of non-renewable energy consumption on human health, there is a need to develop sound social, economic, environmental, and health policies at Asia's national and sub-national levels. Findings recommend that there is a need for national and international agreements to move economies from dirty energy consumption to clean energy sources to help address these serious health issues. Asian peoples should also use clean energy technologies for the environment and good health. Green investment in clean infrastructures is heavily required, particularly in Asia, where improves human health. The Asian governments should make efforts to increase the investment in the health sector.

A limitation of the study is that we could not include disaggregated data within the energy due to the unavailability of data for a long period of time. The study could not measure the impact of renewable energy consumption on health outcomes in Asia. We could not consider moderate factors that directly or indirectly affect health outcomes due to energy consumption. Authors should focus on the effect of disaggregated renewable energy consumption on health outcomes in Asia in future studies. Upcoming studies should also incorporate the COVID-19 pandemic in empirical analysis by adding renewable and non-renewable energy consumption to health production.

## Data Availability Statement

Publicly available datasets were analyzed in this study. This data can be found here: https://data.worldbank.org/.

## Author Contributions

XZ: conceptualization, software, data curation, and writing—original draft preparation. MC: methodology, writing—reviewing, and editing. JL: visualization and investigation. All authors contributed to the article and approved the submitted version.

## Funding

This work was supported by the Digital Inclusive Finance and financing constraints of SMEs (ZR2020MG042), Shandong Natural Science Foundation; Research on the effect and mechanism of judicial environment on the recovery of defaulted loans of SMEs (72003110), and Natural Science Foundation of China and Project of Humanities and Social Sciences of Cultivation Plan for 1000 Middle aged and Young Backbone Teachers in Higher Education Institutions in Guangxi.

## Conflict of Interest

The authors declare that the research was conducted in the absence of any commercial or financial relationships that could be construed as a potential conflict of interest.

## Publisher's Note

All claims expressed in this article are solely those of the authors and do not necessarily represent those of their affiliated organizations, or those of the publisher, the editors and the reviewers. Any product that may be evaluated in this article, or claim that may be made by its manufacturer, is not guaranteed or endorsed by the publisher.
